# Neuromorphic hardware for somatosensory neuroprostheses

**DOI:** 10.1038/s41467-024-44723-3

**Published:** 2024-01-16

**Authors:** Elisa Donati, Giacomo Valle

**Affiliations:** 1 Institute of Neuroinformatics, University of Zurich and ETH Zurich, Zurich, Switzerland; 2 Department of Organismal Biology and Anatomy, University of Chicago, Chicago, IL USA

**Keywords:** Biomedical engineering, Somatosensory system

## Abstract

In individuals with sensory-motor impairments, missing limb functions can be restored using neuroprosthetic devices that directly interface with the nervous system. However, restoring the natural tactile experience through electrical neural stimulation requires complex encoding strategies. Indeed, they are presently limited in effectively conveying or restoring tactile sensations by bandwidth constraints. Neuromorphic technology, which mimics the natural behavior of neurons and synapses, holds promise for replicating the encoding of natural touch, potentially informing neurostimulation design. In this perspective, we propose that incorporating neuromorphic technologies into neuroprostheses could be an effective approach for developing more natural human-machine interfaces, potentially leading to advancements in device performance, acceptability, and embeddability. We also highlight ongoing challenges and the required actions to facilitate the future integration of these advanced technologies.

## Abstract (web summary)

Neuroprosthetic devices have recently emerged as promising solutions to restore sensory-motor functions lost due to injury or neurological diseases. In this perspective, Donati and Valle propose to combine neuroprostheses with neuromorphic technologies for designing more natural human-machine interfaces with possible improvements in device performance, acceptability, and embeddability.

## Opportunity and challenges

Neuroprosthetic devices have been recently proposed as promising solutions for restoring sensory-motor functions lost after injury or neurological disease, as described in Box 1. These devices extend implantable neural interfaces, which establish a functional connection pathway between the human nervous system (e.g., peripheral somatic nerves, cervical or lumbar spinal cord, or somatosensory cortex), and digital or robotic technology (e.g., computers, prostheses, or robotic devices). Similarly, electrical neurostimulation has been demonstrated to be a powerful tool for restoring sensory feedback in people with sensory loss (e.g., amputees or individuals with spinal cord injury). In contrast to purely motor neuroprostheses (such as those for restoring locomotion^1^, speech^2^ or hand functions^3^), sensory neuroprostheses require a bidirectional loop between the brain and the robotic device with both volitional control and sensing. Sensory neural interfaces require not only the ability to record, but also to deliver microstimulation selectively in order to activate the neural tissue; algorithms for controlling the multidimensional space of stimulation parameters in an effective and efficient manner; and wearable sensing technology for detecting body–environment interactions. However, restoring any sensory information is extremely complex due to its multidimensionality, both in time and space. Restoring somatosensory touch feedback requires sophisticated stimulation strategies that simultaneously modulate multiple parameters, e.g., active channels, pulse frequency, pulse amplitude, and pulse width. This results in restored sensory information which is still limited compared to the natural sense of touch. Multiple challenges are currently limiting the adoption and applications of somatosensory neuroprostheses in the clinical sphere. The main areas of current development include (1) neural interfacing (including material, biocompatibility, efficacy, etc.); (2) algorithms for decoding neural signals and encoding artificial sensations (e.g., real-time bidirectional systems, AI-based decoders, and biomimetic encoders); and (3) the engineering challenges of developing the hardware to perform and apply the necessary computations (e.g., wearable devices, fully implantable systems). Considering that presently there are electrodes implanted in humans, both in the PNS^4,5^ and CNS^6^ which have endured for many years, it is possible to develop and test novel algorithms for improving the efficacy and functionality of these devices. Recently, biomimetic neurostimulation that simulates the natural touch coding with in-silico models has been proposed as a promising approach to conveying sensory information in hand and leg prostheses and has been tested in first-in-human trials^7–11^. However, current state-of-the-art methods for obtaining realistic modeling simulations of the natural sensory processing, based on detailed biophysical descriptions, use computationally expensive processes implemented on external controllers which are connected to complex neurostimulators that cannot yet be embedded into implantable devices^12–14^. Translation of this approach into clinical practice would require implementing real-time algorithms that meet the hardware constraints of portable devices.

**Fig. 1:**
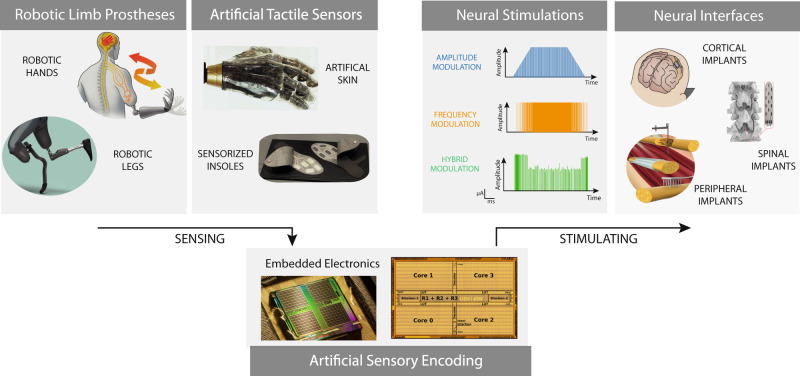
Neural prostheses for sensory feedback restoration. The main building blocks of a neural prosthesis for the somatosensory system are the sensing block, the computing block, and the stimulating block. Sensing technology (e.g., wearable tactile sensors) has to be embedded in the robotic prostheses in order to extract all the relevant physical interactions with the external world. Then, the computing block, composed of the neuromorphic technology, has to translate the sensors’ readouts into electrical patterns of stimulation using biomimetic encoding algorithms. Finally, the stimulating block has to inject currents in the nervous tissue, through implantable electrodes, able to evoke natural neural activations, allowing for natural and informative sensations. A portion of the illustration is adapted from ref. ^32^.

Neuromorphic technology provides alternative design solutions that present a paradigm shift where, rather than simulating the complex biophysics of mechanoreceptors, their behavior is faithfully emulated through the physics of electronic circuits. To achieve the restoration of the somatosensory experience, the neuromorphic system can translate sensory information, recorded by wearable sensors embedded in the prosthetic limb, and generate biomimetic neural stimulation patterns (Fig. 1). Contrary to traditional digital processors, this neuromorphic approach employs analog/digital mixed-signal CMOS-based hardware that supports in-memory computing and the implementation of neural computational primitives inspired by neural circuitry, such as biological time constants and adaptation at different time scales^15^. Neuromorphic computing has been extensively used for processing physiological data and generating actions, both in the domain of prosthetic control^16,17^ and bioelectronic medicine^18,19^. However, in the domain of somatosensory feedback restoration, neuromorphic hardware implementation has mainly focused thus far on the design of event-based sensors able to encode touch information in trains of spikes^20^, and/or in simulations of spiking neurons using traditional digital processors or field-programmable gate arrays (FPGA)^21,22^. In addition to using standard CMOS technology, there are several attempts at implementing neural interfaces using stretchable^23^, magnetometric^24^, and organic electronics^25^ with the goal of reproducing sensory feedback. Using soft electronics to build artificial skin brings several advantages in terms of tissue conformability, minimal invasiveness, and unobtrusiveness that increase the subject’s engagement^26^. However, to fully deploy this new technology, existing soft electronics require overcoming several challenges in biointegration, low power working regimes, and circuit complexity. Despite the current promising attempts (see Box 2 for more details), neuromorphic technologies still have a long way to go and could be further developed to increase the complexity of the modeling and include more mechanisms present in the afferent pathway, increase the degree of similarity with biological systems, and provide an approach that will revolutionize the field of neuroprosthetics. This new advancements will enable multiple benefits for users, including improved sensation naturalness, prosthesis embodiment, and home-use functionality. In addition, this approach will bring advantages in information encoding, providing an energy-efficient, compact, and embedded solution. However, there are still open challenges for this approach that range from the availability of a full neuromorphic pipeline (sensor–processor–stimulator) to the design of a proper interface for the generation of the actual pattern of stimulation, up to its translation in clinical trials.

In this perspective, we report existing works based on neuromorphic closed-loop systems, which are designed for sensing and controlling prosthetic devices through direct interfaces with the human nervous system in chronic and incurable neurological conditions^27–29^. We present the required steps to build the next sensory feedback for limb prostheeneration of neuromorphic hardware for restoring sensory feedback in neuroprostheses; as well as discuss the combined challenges of neuroprosthetic devices and neuromorphic technologies, suggesting possible research directions to overcome the current roadblocks and enable more natural human–machine interfacing.

Box 1. Prosthetic hand control Bidirectional prostheses are closed-loop technologies with two-way communication to and from the nervous system in which the user both perceives sensations through artificial stimulation and drives a robotic arm through the exploitation of their residual muscles and nerves^4,39,40^. Despite recent advancements, current solutions for the bidirectional control of hand prostheses still have non-trivial limits which reduce the overall usability of dexterous hand prostheses. Dexterous prostheses are controlled by processing electromyographic (EMG) signals recorded from the residual muscles of the amputee in the stump or elsewhere. Surface EMG (sEMG) acquisition does not require surgery, so it is largely used in upper-limb prosthetic control^154^. However, despite the accessibility, EMG control provides limited usability in practice due to high degrees of freedom and is further limited when external factors are introduced, such as changes in electrode position or environmental conditions^155^. The most common control approach is based on decoding EMG signals using traditional machine learning approaches (e.g., MLP, SVM) to discriminate users’ gestures. However, despite the remarkable results, the identified gestures are rather simple and discretized, rendering the control less natural whilst requiring high power consumption^39^.

Improvements can be achieved by increasing the invasiveness of the approach, depending on the specific type of amputation. For high-level amputations, Targeted Muscle Reinnervation (TMR)^156,157^ can provide a suitable surgical technique to enable prosthesis control using EMG signals. Once the residual nerve is reinnervated to another muscle, it works as a biological amplifier providing appropriate EMG signals for motor commands. For trans-radial amputees, epimysial electrodes showed improvements in prosthesis control with respect to sEMG^158,159^ in terms of robustness to environmental conditions. Moreover, recent approaches have been developed to extract the sources of neural information through EMG deconvolution, exploiting advanced multi-channel EMG systems^160–163^.

Increasing efforts have also been carried out on the development and use of neural interfaces able to record electroneurograms (ENG) related to hand motor commands from the residual nerves of amputees^70,164^, or to focally amplify signals through surgical procedures (AMI^41^ or RPNI^36^). Thanks to the intimate interfacing with the residual nervous system, intraneural electrodes have the potential to record from different efferent fibers and fascicles, allowing the identification of a large set of volitional motor commands. In particular, the extraction of informative neural signals related to motoneurons could be challenging since sensory axons, innervating the human arm, outnumber motor axons by a ratio of at least 9:1^165^. Thus, intraneural or intrafascicular interfaces could provide more selective contact with motor fibers than less invasive approaches, allowing for an improved signal-to-noise ratio. However, this approach is still far from being implemented in a real-life clinical setting.

Regarding the decoding algorithms for clinical application, prosthetic control can be divided into three main areas:^166,167^ (i) simple proportional control, where the amplitude of the remnant muscles’ activity is mapped to the hand’s force or speed; (ii) pattern recognition, where the system produces a discrete movement from a predefined set; (iii) regression-based algorithms able to continuously estimate multiple control signals. The current state-of-the-art in prosthetic control is based on pattern recognition using machine learning^168^ or deep learning^169–171^. However, a major disadvantage of a machine learning pipeline is the strong reliance on domain-specific knowledge, needed to properly choose features to feed the machine learning algorithm (feature extraction), select the best ones among them (feature selection), and tune the hyperparameters of the target algorithm. Although deep learning loosens these requirements by replacing feature extraction and selection with feature learning, incorporated into the algorithm’s training, the system requires large datasets that are difficult to generate and is not suitable for continuous motor discrimination.

In recent years, regression-based algorithms have shown a strong potential to deliver robust prosthetic control^155^. Such methods enable simultaneous and proportional control by modeling the relationship between an EMG and the kinematics^172^ or dynamics^173^ of the gesture and achieve more intuitive control of the prosthesis^174^. Regression captures the temporal nature of the EMG, yielding more natural and versatile control. However, a substantial limitation of the cited contributions is that the issue of implementation on embedded/wearable computational platforms is not addressed, because they were not designed to deal with resource constraints, such as memory, power, and latency, required for real-time execution in daily use.

Box 2. State-of-the-art in neural prosthetics Neural prosthetics is a category of human–machine interfaces that aim to replace motor, sensory, or cognitive impairments due to injury or disease. Among them, the most advanced are currently visual and cochlear implants.

Cochlear implants are surgically implanted neural prosthetic devices able to restore the loss of hearing functions by stimulating spiral ganglion nerves. The implant consists of an array of electrodes implanted along the cochlea such that each section stimulates a region corresponding to a specific frequency^175^. Most control strategies are based on amplitude modulation of a biphasic current pulse train^176^. However, recent work uses bio-inspired coding strategies, based on the electrically evoked compound action potentials, showing a better representation of spectral and temporal information^177^. In neuromorphic computing, the research focuses on the development of event-based cochlea using CMOS^178^, FPGA^179^, or neuromorphic microelectromechanical systems^180^ and has not yet explored neural prostheses.

Visual implants are composed of an external camera that captures the visual information and sends it to a signal-processing unit for conversion into a sequence of stimulation patterns which are then sent to the stimulation electrodes.

Depending on the approach of stimulation there are four main locations^181^: 1. retinal^182^; 2. lateral geniculate nuclei of the thalamus (LGN)^183^; 3. optic nerve^184^; and 4. cortical^185^. Recently, works based on the neuromorphic implementation of silicon retinas have been proposed to develop a new generation of neural prostheses^186–188^. Compared to standard cameras they show improved perceived light dots (phosphenes) in difficult lighting conditions and with motion blur^189^.

Despite the recent progress, the sensation elicited by current devices is perceived as artificial and distorted. Therefore, the identification of the effect of stimulation on the nervous system can lead to disruptive results in the field of neuroprosthetics. An example in visual implants shows that varying the stimulation waveform manipulates the response of the retinal ganglion cells, by changing the threshold, which could potentially lead to a higher spatial resolution in shape perception^190^. Another aspect to consider is the spread of activation of each electrode since it is not point-like, and it may introduce overlap with activations from other electrodes. This drawback can be solved by using “shaping” algorithms based on multiple electrodes able to manipulate the current in a desired way^191^. Among the various strategies to generate a proper stimulation strategy, there are approaches based on deep neural networks used to build an end-to-end neural prosthesis^192^ that can also be trained to approximate the underlying biological system^193^. In addition, convolutional neural networks are also used to predict the electrode activation patterns required to generate a desired stimulus in visual perception^194^.

## Requirements for neuromorphic hardware for somatosensory neuroprosthesis

Combining advanced sensing systems with neuromorphic encoding schemes and novel neural interfaces would allow for highly functional sensory neuroprostheses, a higher sense of embodiment, and a more complete integration in the human body schema. A closed-loop neuromorphic prosthesis would also provide benefits in terms of information bandwidth, parallelization, and portability, allowing for improved biomimetic feedback.

## Interfacing to the human nervous system

### Neural interfaces for communicating with the brain through neurostimulation

The field of implantable neural interfaces has witnessed rapid progress in the past 30 years^30–32^. Indeed, several breakthroughs in the interfacing of the nervous system and electronics have been achieved, many of which have clinical relevance. When entering the phase of clinical application, it is of paramount importance to understand the working mechanisms of neural stimulation and to develop fully integrated neurotechnology able to effectively replace or repair as many biological functions as possible. This can substantially improve the quality of life of patients with sensory-motor deficits.

Different types of neural interfaces have been adopted to record the neural activity from the brain^33–35^ or nerves^36–38^ and, more recently, to also deliver electrical stimulation to peripheral nerves^4,11,39–44^ (see Box 3 for more details), spinal cord^45,46^, or cortex^47–49^ to restore sensory-motor functions. The use of electrical stimulation patterns allows for a valuable and reliable tool to directly communicate with the human nervous system enabling it to encode artificial sensory information^50^. Its effectiveness and functionality for sensory feedback restoration are strongly dependent on the characteristics of the adopted neural interface (i.e., implantable electrode). Indeed, the implanted electrode should be highly: (1) biocompatible; (2) selective; (3) modular; and (4) stable.

Biocompatibility is defined by the intrinsic properties of an electrode such as the materials it is made of, its size, and the implantation procedure. A biocompatible interface should be made with certified and standard materials for active implantable medical devices as required for clinical application^51^. Another important factor affecting biocompatibility is the difference in Young’s modulus between the electrode and the nervous tissue. The smaller the difference, the less the mechanical stress of the interface on the biological tissue^52^. Also, the size ratio between the electrode and the neural tissue could affect its biocompatibility. Smaller electrodes may have less impact on the biological environment potentially improving the integration of the device in the body, but at the expense of robustness and maximum injectable charge. Finally, the level of invasiveness of the implantation substantially influences the foreign body reaction (FBR) or inflammatory response that can be responsible for electrode failures or a decrease in their effectiveness^53,54^. Notably, recent results have demonstrated that non-invasive technologies using remapped evoked sensations are less informative, and intuitive than the neurally evoked somatotopic sensations restored via implantable devices^55,56^.

Electrode selectivity is currently of great interest to many research groups. Indeed, selective stimulation (i.e., defined as the capability of the electrode to activate small groups of neurons without unintentionally activating other neural regions) could allow substantially improved effectiveness of these interventions, minimizing the side effects. The selectivity is dependent on electrode size, shape, active site configuration, and stimulation protocol. In general, electrodes penetrating the nervous tissue (such as intraneural or intracortical) allow for more intimate contact of the electrically active sites with the target neurons. This scenario, together with a smaller active site area compared to extraneural or epicortical electrodes, guarantees a more focal stimulation, activating fewer neurons simultaneously. Moreover, electrodes with multiple active sites and also with multipolar configurations are able to shape the electric field achieving more selective stimulation^57^. Thanks to the high selectivity, the stimulation serves its intended purpose with reduced side effects. More recently, stimulation protocols co-modulating injected charge and high frequency could potentially increase the selectivity of the adopted interface and therefore its effectiveness^58^.

In addition, electrodes allowing for high maximum injectable charge and stimulation frequency, yield a wider space for parameter modulation. This is of crucial importance for developing more sophisticated stimulation strategies allowing the encoding of more complex features of the sensory experience. A modular interface exploits specific active site materials and coatings to improve injectable charge limits^32,59^.

The purpose of using implantable neural interfaces is to obtain an effective neural link that can be exploited for long-term applications in everyday life. To this aim, the interface should remain stable and reliable over time. Notably, the stability is strongly dependent on the electrode materials, shape, and implantation procedure. The invasiveness of the implant, in this case, decreases the potential stability of the electrode since the FBRs and the electrode migration are more likely to modify the electrode–neural tissue interface^32,60^. However, the majority of the implants adopting penetrating electrodes, in particular in Brain–Computer Interface (BCI) applications, lasted for several years in human trial applications (more than 10 years)^6,61^. Nevertheless, novel electrode materials and coatings have the potential to improve the long-term stability of intracortical and intraneural interfaces^62^.

Box 3. Neural interfaces for peripheral nerve interfacing Recently, technological breakthroughs in nerve–machine systems were translated into the language of the nervous system, and delivered via electrical stimulation to the residual nerves (PNS) or central nervous system (CNS) of neurologically disabled individuals^195^. In current neuromodulation devices, the design and material of the neural interface are crucial for their wider adoption and exploitability^32^. An adequate neural interface should be able to create selective contact with different fascicles in the nerve bundles to restore the efferent and afferent neural pathways effectively. To achieve a selective electrical interface to the PNS, various types of microelectrodes have been developed and used over the last years^32,196^. The design usually depends on the respective application. Often, very small structures are required to meet the high demands on selectivity and to address very small regions of neural tissue, e.g. individual fascicles^36^. For this reason, these small electrode structures are usually fabricated by microtechnological processes, e.g., the creation of silicon-based structures, such as Utah arrays^197^. These electrodes have been exploited in cortical implants both in animals and, more recently, also in humans. Indeed, Utah arrays are efficient for intracortical implants, where they are anchored to the skull with pedestals and screws. For this reason, they are still the most adopted technology for implantable BCI applications^33,34,47,48,61^. However, for PNS interfacing, the drawback of using silicon-based electrodes is the mismatch between the mechanical properties in the implant and the nerve tissue, leading to increased stress, tissue damage, and encapsulation of the implant by a layer of connective tissue^198^. For this reason, the development of flexible thin-film electrodes based on polymers such as polyimide^199^ represents a promising solution. For the electrode conductive tracks, noble metals such as gold or platinum are typically used to improve their electrochemical properties; the individual electrode contacts can be coated with functional materials (e.g., IrOx or PEDOT)^23,200^. On the stimulation side, the use of functional coatings could increase the maximum injectable charge at the active sites. Use of iridium oxide as the stimulation contact material with its high charge injection capacity keeps the stimulation sites well in the chemically safe charge injection regime^201^. Indeed, the optimal neural interface should have small enough active sites to deliver repeated and selective electrical pulses, but at the same time have the capability of injecting enough charge to active neurons far away or even with fibrotic tissue in between. Interestingly, the use of graphene could overcome several limitations of current neural probes. It promotes high neuronal affinity, chemical inertness, antioxidation, and anticorrosive properties, optical and magnetic (MRI) transparency, and flexibility, all while remaining highly conductive^202^.

The neural interface electrode has long been the limiting technological component for achieving a successful interface to the nervous system. The current state-of-the-art on implanted interfaces for the peripheral nerve are divided into two types: extraneural, implanted around the nerve trunk, and intraneural, which penetrates the nerve trunk. Extraneural cuff electrodes are reliable and robust^4,203^ and imply reduced invasiveness, but suffer from limited selectivity^204^ and limited capability to record neural signals. For improving selectivity, intraneural electrodes that are inserted longitudinally (LIFE^44^) or transversally (USEA^11^, TIME^9,39^) into the peripheral nerve has been developed and tested in human trials. This approach seems very promising because it combines acceptable invasiveness with good selectivity^60^. However, USEAs are rigid silicon structures that record from the tips of the needles. They are transversally inserted in the nerve causing nerve damage in chronic implants^54^. On the other hand, the state-of-the-art polyimide-based thin-film electrodes are associated with a major difficulty in approval as an Active Implantable Medical Device (required for clinical application), because they employ non-standard materials (polyimide)^201,205^. In addition, the longevity and stability of these implants have not yet been fully demonstrated in long-term clinical trials (no longer than 6 months^39,60^). Recently, to achieve both greater signal specificity and long-term signal stability, the regenerative peripheral nerve interface (RPNI) has been developed as a promising solution. An RPNI is composed of a transected peripheral nerve, or peripheral nerve fascicle, that is implanted into a free muscle graft^206^. The free muscle graft undergoes an approximately 3-month process of revascularization, regeneration, and reinnervation by the implanted peripheral nerve. This generates a stable, peripheral nerve bio-amplifier that creates high amplitude EMG signals which can be used to control a prosthetic device^36,207,208^. Moreover, RPNIs have been shown to prevent and treat neuroma pain and phantom pain after amputation^209^. Finally, organic materials have also been proposed as promising candidates for neural interfaces, due to their mechanical softness, excellent electrochemical properties, and biocompatibility. In addition, organic nervetronics, which mimics functional properties of the biological nervous system, has been developed to overcome the limitations of the complex and energy-consuming conventional neuroprosthetics that limit long-term implantation and daily-life usage^210^

### Biomimicry as a solution for restoring naturalistic sensation

The human skin is an incredibly complex and sophisticated deformable organ capable of sensing any type of mechanical interaction of the human body with the environment. For instance, the skin on our hands is innervated by tens of thousands of mechano- and proprioceptive receptors, each of which carries different (albeit overlapping) information on items that are being gripped^63^. This neural information is then processed at multiple levels along the somatosensory axis from the dorsal root ganglion (DRG) to the cuneate nucleus, thalamus, and then to the primary somatosensory cortex^64^. From the idiosyncratic spiking responses of each of the afferents, the tactile information is processed and becomes a conscious percept. To artificially communicate with this complex system, a detailed knowledge of the neural geometry of touch is of crucial importance. Indeed, restoring natural touch through neural interfaces would require stimulating each of these sensory neurons independently with their own idiosyncratic activation patterns, in a way that current technologies are nowhere near ready to implement. State-of-the-art neural interfaces have tens or hundreds of stimulation channels^31^, not the thousand and more that would be required for full biomimetic restoration of touch on the palmar surface of the human hand. Moreover, each electrode activates tens or hundreds of axons and elicits highly unnatural synchronous activation of these afferents. Until much denser and more selective neural interfaces become available, attempts to mimic natural nerve responses will have to settle for mimicking aggregate neural responses^14,50,65^. In the natural sensory processing condition, the spatiotemporal dynamics of this aggregate population response in the sensory cortex seem to mirror those of the population response in the nerves^66^, making the assumptions more generalizable for the entire somatosensory neuroaxis. Moreover, considering that the natural sensory system is encoding an enormous amount of information about the tactile experience (such as intensity, type, location, temperature, and other characteristics of the tactile stimulus), one way to mitigate the complexity issue is to translate this information into the artificial sensory space exploiting biomimicry^67^. Indeed, this method exploits, as far as possible, existing perceptual representations of natural sensory processing. In fact, to the extent that we can reproduce the neuronal firing patterns that would occur with an intact nervous system, the resulting perceptions could be more natural^9^ and would also require minimal learning. While we cannot completely reproduce natural patterns of neuronal firing, we can exploit key principles of sensory processing in the development of the encoding algorithms^7,8^. In particular, the spatial and temporal components of the neural activation could be modulated and partially controlled through the neurostimulation parameters^8,68^. This would allow the exploitation of residual sensory processing for evoking natural and informative percepts.

**Fig. 2:**
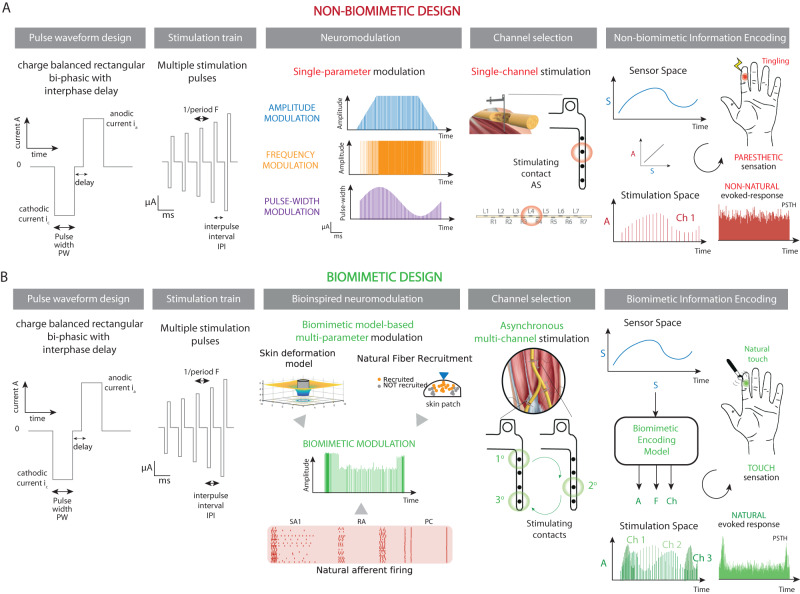
Biomimetic approach for encoding sensory information via neural stimulation. **A** The whole pipeline of neural stimulation patterns for somatosensory feedback restoration based on non-biomimetic design and **B** based on biomimetic design. In the latter case, the stimulation parameters, such as frequency, amplitude, and stimulating channels are modulated in order to evoke natural patterns of neural activation. A portion of the illustration is adapted from ref. ^32^.

Currently, in the neuroprosthetic field, the scheme of neural stimulation is mostly not defined by the nerve’s natural coding or neuromorphic models (Fig. 2A)^50^. Indeed, after defining the pulse waveform of the stimulation train, the modulation occurred only on a single parameter and mostly on individual channels of the neural interface. This unnatural approach causes the evoked sensations to be mostly described by users as vibration, tingling, paresthesia, or electricity^32^. In fact, paresthetic sensations are likely to arise from the unnatural activation of nerve fibers^58^ and can be due to the over-excitation of afferents or cross-talk between them^69^. The encoding functions that are used to modulate the injected charge (pulse width or pulse amplitude)^4,39,40^ or pulse frequency^70,71^ of the delivered neurostimulation pulse–train according to values read from the prosthetic sensor. This mapping generally follows a linear and proportional relationship to the sensor readings (e.g., the higher the value of the pressure measured by the sensor is, the higher the stimulation charge will be). Therefore, the intensity of the perceived sensation is proportionally associated with the stimulation charge and frequency (the higher the injected charge is, the higher the perceived sensation intensity will be)^47,72^. Interestingly, the use of sinusoidal modulation of the stimulation pulse width has shown promising results in improving sensation quality^40^. Unfortunately, these findings have not yet been replicated by other research groups^73^. The sensory transformation from paresthesia to natural qualia seems to require more than patterned and sinusoidal neurostimulation.

In fact, natural touch coding, and the relationship between biological sensors (cutaneous receptors) and neural activity is more complex than a sole single-parameter coding. For example in natural touch, the information about transients is much more salient and prominent in the neural code than the information about sustained stimulation^63,66^, as it includes both rate and temporal coding. Thus, a more biomimetic pattern should incorporate this information, highlighting the contacts^7^, and should modulate multiple parameters and electrodes at the same time^7,9^. The target aim is to electrically induce a natural pattern of neural activation^67^, such as the one generated by the biological receptors in the case of healthy touch systems. The theory of adopting more biomimetic and bio-inspired patterns of stimulation assumes that replicating the natural firing patterns would lead to more natural and intuitive sensations (Fig. 2B). The spatio-temporal modulation (multi-parameter and multi-channel) of the neural stimulation would allow to generate more complex and natural evoked activity of the recruited neuron population. To do so, computational models^12–14^, able to replicate the natural neural responses, could be adopted. To develop such models is fundamental to have ground truth of the natural spatiotemporal activation that the artificial system needs to replicate. This will require models able to run in real-time and able to generalize to every type of tactile input coming from the external environment. Neuromorphic technology can provide systems able to emulate (with low power consumption) the natural neural responses of different neurons/networks and also to learn and adapt to different tactile inputs.

## Neuromorphic technology in neuroprosthetics

### Neuromorphic hardware to boost neuroprosthetic applications

Neuromorphic technologies use event-driven, parallel, and in-memory computing techniques to emulate computations performed by biological nervous systems^74,75^. They comprise of neuron and synapse circuits implemented using standard CMOS technologies with biologically plausible time constants (from milliseconds to seconds or even minutes) that make them ideal candidates to emulate neural dynamics^76^. Unlike conventional digital technologies, neuromorphic processors implement memory storage co-distributed with the processing, which allows memory and power consumption to be minimized^77^. Moreover, they often support network reconfigurability^17,78^, conveying information as temporal spikes rather than numeric values^79^. Spikes allow sparse and asynchronous communication which relies on the time of firing and enables efficient spatiotemporal pattern discrimination^80^. Box. 4 outlines the features of some representative neuromorphic processors.

In the field of neuroprostheses, neuromorphic solutions are increasingly explored to build tactile sensors and restore feedback sensation. In the past decade, different ways to create an artificial sense of touch have been explored using different sensors, e.g., resistive^81^ piezoresistive^82,83^, capacitive^84^, optical^85^, piezoelectric^86,87^, or acoustic^88^. As in biology, event-driven sensors locally encode changes of stimulus intensity with a train of spikes utilizing a specific dynamic temporal pattern, in contrast to conventional tactile sensors that are sampled periodically and return a continuous scalar pressure value even when no force is applied. Encoding changes decrease the data to process, allowing a more energy-efficient and biomimetic approach. Current implementations of neuromorphic tactile sensors can be divided into two classes: soft neuromorphic, where tactile sensors are combined with a microcontroller or other digital processors to simulate spiking model neurons, or neuromorphic sensors that directly output spike trains.

In soft applications, the sensor outputs are transmitted into the digital processor or FPGA where a neuron spiking model encodes the signal into spikes^21,22^. Although these implementations are convenient solutions for system-level applications, the need for a digital device to simulate simple spiking neuron models limits the power performance and miniaturization. This approach allows the implementation of multisensory systems with high spatial resolution. An example includes a 64 × 64 resistive sensor matrix combined with a piezoresistive fabric with a sub-millisecond response time and a sampling frequency of 5 kHz. The sensory array is connected to an FPGA to handle the parallelism of the sensors and to generate spikes every time a significant change is detected for a particular element^89,90^. Another implementation is composed of two by two microelectronic mechanical systems (MEMS) arrays, where each sensor is composed of 4 piezoresistors working at 375 Hz interfaced with an FPGA that simulates a simple model of spiking neurons, the Izhikevich model^91^, to reproduce the behavior of tactile receptors^22^ which can be applied to the categorization of naturalistic textures^92^. The asynchronously coded electronic skin (ACES) consists of 240 artificial mechanoreceptors that asynchronously transmit spikes with a latency of 1 ms and an ultra-high temporal resolution precision of <60 ns. Each receptor consists of a resistive sensor, a microcontroller for the generation of the spike, and a series of passive components to perform signal conditioning^93^. Soft implementations are used for both stimulations, mainly non-inasively^21,94,95^, and, more extensively, for texture discrimination. The feasibility of texture discrimination has been demonstrated using different approaches, with the most notable approach using piezoelectric sensor arrays and the Izhikevich spiking neural model. Various analytical methods were applied for the investigation of spatiotemporal spike patterns, such as spike train distance-based kNN^96^, interspike interval statistics and spike distance metrics^92^, a recurrent spiking neural network^97^, and an extreme learning machine^98^. Another approach is based on 24 capacitive square sensors in a rectangular grid layout working at 20 kHz. The output is proportional to the intensity of the force and it is converted into spikes using an integrate and fire neuron utilizing an adaptive threshold. This has been used for the classification of Braille stimuli^99^. However, while these implementations can serve as tools for the development of applications, they do not reach the same low-power performance and miniaturization of directly designing biomimetic sensors, and the neural model investigated is rather simple, although other neuron models can be explored.

Current neuromorphic implementations based on mixed-mode subthreshold circuits represent promising solutions to solve the problem of power consumption and miniaturization. However, there is the need for multiple transducers to emulate different mechanoreceptors, and the difficulty of their mechanical interfacing limits their stable integration on prostheses or robotic platforms^100^. They include piezoelectric transducers^99,101,102^, which detect fast changes, and hence emulate the behavior of rapid adapting mechanoreceptors (RAs), and capacitive transducers which have the advantage of keeping their capacitance over time enabling the emulation of slow adaptive mechanoreceptors (SAs)^103^. Another example uses semi-volatile carbon nanotube transistors to build a sensor system with sensory neurons and a perceptual synaptic network to differentiate the temporal features of tactile patterns^104^. In both implementations, the silicon neuron is a LIF neuron with the basic neural function of integrating the incoming information and encoding the information into a spike once the membrane voltage reaches a threshold.

At present, neuromorphic implementations are still in a proof-of-concept stage, and they have not yet been tested in neuroprosthetic applications. However, they have been deployed for spatiotemporal discrimination tasks, such as Braille letter reading^105^. Very recently, Wang and colleagues presented a promising monolithic soft prosthetic e-skin capable of multimodal perception, neuromorphic pulse-train signal generation, and closed-loop actuation. With a trilayer, high-permittivity elastomeric dielectric, they achieved a low subthreshold swing comparable to that of polycrystalline silicon transistors which operate at a low voltage, consume little power, and integrate medium-scale circuits that enable stretchable organic devices^26^.

A third explorable alternative is the integration of standard sensor arrays with neuromorphic technologies, where the mechanoreceptors’ behavior is not simulated in a digital processor but emulated in silicon neurons, and their behavior is set by the neurons’ parameters. This approach replaces the digital processor of the soft solution with a more power-efficient solution; however, it adds more circuit complexity than that present in event-based pixels. The adaptation in the mechanoreceptors can be emulated by a non-linear adaptive neuron circuit with a calcium channel responsible for the spike frequency adaptation in real neurons^106^. The silicon neuron model can vary from a complex conductance-based Hodgkin–Huxley model with a high degree of biological compatibility^107^, to the Adaptive Leaky Integrate-and-Fire (AD-LIF) model. Recent implementations of AD-LIF silicon neurons use 22 nm technology, with a layout area of 15 × 60 μm^2^ per neuron, an adaptation time constant of up to 5 s, and a power consumption of 14 pJ/spike, that enables its use in edge computing applications, including integration in neurorobotic applications^108^.

Compared to standard approaches, neuromorphic tactile sensors offer a solution to the drawback of limited bandwidth, both in terms of the frequency of the acquired data and the high-density receptor arrays, by using populations of spiking neurons with limited bandwidth (e.g., able to fire at most at a few Hertz or tens of Hertz) that can collectively encode signals with much higher bandwidth and dynamic range^109^. Sensors could transmit the generated spikes by using the address event representation (AER) communication protocol, which is the most common protocol in neuromorphic technologies, and utilizes the address of the source or destination neuron to distinguish the spikes that travel on a shared digital bus^110^. To further improve the distributed sensory transmission, a compressive method on the sensor side using asynchronous impulse radio ultra-wideband (IR-UWB) for wireless low-power communication can be explored^111^. Wireless communication using IR-UWB could improve system acceptance and usability reducing the use of wires that pass along the prosthetic device.

#### Pattern generation for electric stimulation

Once appropriate mechanoreceptor output spike trains are generated, they serve as the input to generate the activation pattern for electric stimulation. Generating an effective natural pattern requires a compression stage and a transformation in the stimulator’s parameters, combining multiple spike trains into a single train for use in clinical applications. Common approaches include computing the sum (representing an aggregate neural response) or the average of the output spike trains^9^, which may result in a high firing rate where spatial information could be compromised. In traditional machine learning, the use of compression algorithms, such as clustering and classification, are utilized to extract important features in real-time applications^112^. In cortical recordings, multiple spike trains can be merged into a single spike train by identifying temporal spike patterns within mechanoreceptors’ spike trains by analyzing temporal correlations with statistical methods such as entropy, or cross-correlation^113^. In neuromorphic sensors, while some studies address temporal compression^114^, only a few have focused on spatial and temporal down-sampling on event-based data, primarily relying on hardware filters and focusing on event-based cameras^115,116^.

To date, only soft neuromorphic solutions have been interfaced with non-invasive stimulation through transcutaneous electrical nerve stimulation (TENS)^94,95^, where the number of spikes is calculated at each time point and a threshold is set to define the sensitivity and accuracy of the spiking behavior^21^.

An unexplored approach is the use of a recurrent neural network that receives the mechanoreceptors’ output as its input and analyzes sequences by storing information in its internal memory state and outputs a singular pattern^117^. Although the network benefits from potential implementation on a neuromorphic chip, its implementation is not straightforward since it would require an appropriate training function, similar to autoencoders, where the compressed information is used to recreate the original signal. In addition, to overcome the problem of misclassification or regression error, we can add a safety mechanism at the output of the network to check the range of the rate of stimulation, avoiding the generation of an unpleasant pattern of stimulation. This safety mechanism can be implemented by an additional layer of recurrent inhibition that compresses the firing rate in a signal with a high dynamic range^118^ or by exploring a new approach of using hierarchical reservoir networks which allows networks to function across markedly diverse timescales, exceeding the performance achievable by single reservoirs^119^.

The compressed spike train is then used as input for the electric stimulator where the spikes are converted into pulses in real time. This conversion can introduce some drawbacks such as delays from the sensor’s output to the electrical stimulation^120^, or a lack of dynamics in the stimulation’s parameters (e.g., frequency, pulse width, etc.)^95,121,122^. To preserve the temporal information carried in the spikes and enable real-time operation, the conversion of the signal to spikes is critical^21^. Three strategies have been proposed and are based on prior work that shows biological significance under electrical stimulation for eliciting sensory perception:^72,123^ pulse width modulation, with fixed frequency and the pulse width dependent on the number of spikes per window; pulse frequency modulation with fixed pulses and frequencies proportional to the number of spikes counted in a time-programmable window; and a neuromorphic match where the temporal information is kept in the neuron’s spiking activity. In the latter approach, the neuron model encodes the information in frequency output, resembling natural modulation. Using silicon neurons rather than simulated neurons will allow for the use of the output neurons as pulse generators by adding a pulse extender circuit to the output of the neuron^124^. The pulse extender enables the generation of pulses with settable width, maintaining compact and low power implementation.

So far, the clinical implementations of neuromorphic approaches are limited to soft neuromorphic sensors in non-invasive stimulation, leaving room to explore neuromorphic solutions in neurostimulation for both sensors and processors.

#### Neuromorphic neurostimulator

Invasive closed-loop applications are still based on biomimetic approaches and, currently, neuromorphic approaches have yet to be fully explored. Biomimetic approaches involve the simulation of mechanoreceptors using powerful simulators running on digital processors^12,13,125^; however, when the complexity of the information encoding increases, it becomes computationally expensive and requires bulky stimulators, hindering real-time portable implementations. As described above, neuromorphic technologies promise a solution by using simulators to emulate the mechanoreceptors’ behavior thanks to their intrinsic properties, thus allowing portable solutions. Different mechanoreceptors can be emulated by setting different parameters in the chip, i.e., threshold, and adaptation time constants^126^ to match both slow and rapidly adapting mechanoreceptors (SAs and RAs). Skin deformation can be emulated by implementing mesh networks, in which neurons are connected with their neighbors via weights that depend on the emulated mechanoreceptor type. The higher the contact force, the further the propagation from the contact point.

To design an end-to-end neuromorphic pipeline and exploit all the advantages of the neuromorphic paradigm, the electric stimulator must be seamlessly incorporated. CMOS-based electric stimulators have been recently proposed and are regarded as ideal candidates^127,128^. Peripheral neural stimulation requires biphasic current pulses (from 10 to 1000 µA) to avoid charge accumulation, at the tissue interface, which would otherwise severely damage neighboring tissue. To design efficient peripheral stimulators, in terms of the amplitude resolution of the stimulus, current-mode stimulators are often preferred, although voltage-mode stimulators are very efficient in terms of power consumption^129^. In current-mode stimulation, the amount of delivered charge is set by controllable parameters such as the injected current and the duration of the pulse^128^. The major issue with current-mode stimulation is the high variability of electrode-tissue impedance (from 10 kΩ to 1 MΩ) that can generate a drop in the voltage. To overcome this limitation, the system can operate under a high voltage supply (from 15 V up to 30 V) that can be generated, and controlled, by embedding programmable voltage boosters. In addition, CMOS stimulators can be affected by mismatches that modify the generated pulses. This can be mitigated by different techniques such as blocking capacitors, or by using resistors connected to the terminals^130^. By using the impulse radio ultra-wideband (IR-UWB) the stimulator can be implanted close to the targeted nerve and can receive input from the processing unit, enhancing the embeddability of the system and simultaneously enhancing the comfort and acceptability for the subject^131^.

#### Location of electric stimulation along the ascending pathway

In addition to the many challenges described in tactile feedback restoration, there remains a need to determine the suitable location for electrical stimulation which depends on the severity of the neural injury and the degree of residual function. Stimulation can target either the peripheral or central nervous systems, but with fundamental differences in the information processing, and limitations imposed by the anatomy and physiology of the site^132^. Peripheral stimulation includes stimulation of primary afferent neurons in the residual limb^39,133^ and of the DRG^134^. Targeting primary afferents and the DRG has the advantage of utilizing the distinct behavior of the various receptor types to directly generate complex stimulation patterns. However, since the somatotopy is not guaranteed within nerves, the specific location of the different fibers is unknown a-priori. Thus, very selective interfaces and a long phase of mapping are required for effective targeting of the PNS. Central nervous system stimulation includes stimulation of the spinal cord^45,135,136^, the ventral posterolateral thalamus^137^, and the somatosensory cortex^47,138^. Stimulation at the spinal cord level presents some advantages compared to stimulation in the residual limb, such as more discernable somatotopy, valid for both distal and proximal amputation; as well as more robust implantation procedures. However, the low selectivity of the spinal electrodes affects the spatial resolution of the restored feedback. Finally, direct stimulation of the somatosensory cortex (Brodmann’s Area 1) using intracortical electrodes (ICMS) exploits the somatotopic organization of the sensory representation, and induces less paresthetic sensations when compared to peripheral stimulation. However, since the neurons are organized in networks and columns, ICMS evokes both direct and indirect neural activation, even in nearby areas (e.g., motor cortex—M1). This can complicate the design of closed-loop BCI^10^. Notably, the higher the stimulation location in the somatosensory neuroaxis, the more the mechanoreceptors’ outputs would require transformation; specifically firing depression and signal integration. Therefore, additional neural and synaptic mechanisms, such as spike frequency, short-time depression, and excitatory–inhibitory balance are required. Due to the high flexibility of their parameter and network reconfigurability, neuromorphic technologies could allow for stimulation at any level within the pathway without the need to rethink and redesign the stimulator.

Box 4. State-of-the-art neuromorphic technology Analog (mixed signal) chips. BrainScales2 is a mixed-signal wafer-scale system that runs in 1000×–10,000× accelerated time and implements physical models of neurons and synapses^211^. It comprises 512 neurons and 112k synapses per core with online spike-driven synaptic plasticity (SDSP). Recent benchmarking of this chip has shown that learning corrects for the mismatch (non-homogeneity of devices) present in the analog hardware^212^. Another example is Neurogrid^213^, from Stanford, which implements a scalable analog neuromorphic chip, simulating up to 1 million neurons and 8G synapses using 1800-fold less energy per synaptic activation than a GPU. Following hierarchical distribution and aggregation of spiking events, like the cortical columns in the cortex, Neurogrid greatly minimizes the required wiring and enables it to simulate multiscale neural models in a time and energy-efficient fashion. DYNAP-SE^214^, and the new generation DYNAP-se2, are designed with an end-to-end event-based sensory-motor system in mind. Implementing synaptic and neuronal dynamics using analog circuits with multiple time constants, allows the real-time processing of incoming signals. Braindrop, a 0.85 mm^2^, 4096-neuron, and 64k-synapse, low-power neuromorphic system, was designed with a comprehensive set of high-level programming abstractions and a synthesis procedure for mapping them to mismatched subthreshold analog hardware^215^. For typical network configurations, Braindrop achieves an energy-per-equivalent synaptic operation of 388 fJ. Braindop has been designed specifically as a substrate for implementing the neural engineering framework (NEF).

Analog neuromorphic technologies represent an ideal solution for highly optimized tasks, thanks to extremely power-efficient and fast asynchronous in-memory computing. It can be deployed for next-generation computing and signal processing—for ultra-low-power and real-time applications. Analog neuromorphic technologies are currently innovating processors with analog computational cores and digital interfaces, improving the reconfigurability and the quality of signal transmission.

Digital chips. IBM’s TrueNorth, a milestone in neuromorphic research, is a multicore processor, with 4096 cores, each one having 256 programmable neurons for a total of a million neurons. Each neuron has 256 programmable synapses, giving a total of 268 million, allowing neural network inference workloads at power levels as low as 70 mW^216^. Another chip was developed by Intel, Loihi^217^, and more recently the second generation, Loihi2, with 1 million neurons and 120 million synapses, allows the implementation of more brain-inspired networks using recurrence, precise spike-timing relationships, synaptic plasticity, and sparsity, which solve a diverse range of problems, such as data processing, adaptive control and constrained optimization, with orders of magnitude lower latency and energy compared to state-of-the-art conventional approaches^217^. Another example is Spinnaker 2^218^, an ARM-based processor built with a GF 22 nm FDSOI process, which takes advantage of the run-time adaptive body biasing technology, which can be exploited for cutting-edge power consumption^219,220^. Another chip built using 28 nm FDSOI technology is online-learning digital spiking neuromorphic (ODIN) which consists of a single neuron synaptic core with 256 neurons and 2562 synapses. Each neuron can be configured to reproduce Izhikevich behaviors^91^. The synapses embed a 3-bit weight and a mapping table bit that allows enabling or disabling SDSP locally, thus allowing for the exploration of both off-chip training and on-chip online learning setups. Brainchip’Akida is one of the first commercial neuromorphic chips, and it is available for integration in ASIC products or as a System on a Chip (SoC) product. The SoC is built around a core neural processor comprised of 80 neural processing units, which enables the modeling of 1.2 million neurons and 10^9^ synapses. It supports convolutional neural networks (CNNs) and fully connected networks^221^. Power consumption in standard tasks ranges from 100 μW to 300 mW.

### Biomimetic neuro-robotic devices

The implementation of a natural neurostimulation approach for sensory restoration requires sensitized neuro-robotic prostheses. A prosthetic device will be used in daily life where the user has to accomplish a variety of motor tasks. The natural feedback provided to the user should match the body–environment interactions in terms of (1) time (i.e., real-time feedback; the sensation has to be perceived without delay with the physical interaction); (2) space (i.e. somatotopic feedback; the sensation has to be perceived in the same location of the physical interaction); (3) modality (i.e., homologous feedback; the type of perceived sensation has to match the type of the physical interaction); (4) intensity (i.e., modulated feedback; the intensity of the sensation has to match the intensity of the sensory experience); (5) naturalness (i.e., natural feedback; the evoked sensation has to be almost identical to a sensation experience on an intact hand). When an artificial sensory modality fulfills all these requirements, it can be considered comparable to a natural sensory modality and will provide high prosthesis embodiment and integration^139^.

**Fig. 3:**
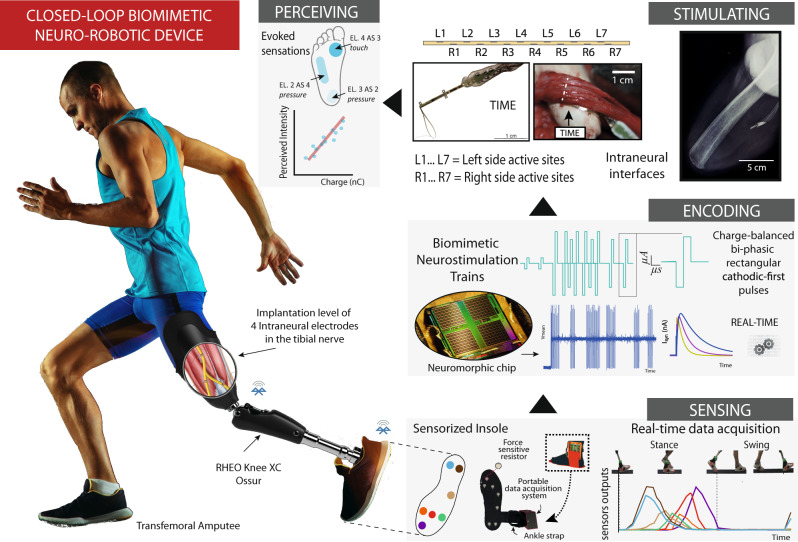
Biomimicry implemented in a neuro-robotic device thanks to neuromorphic hardware. The complete design of a closed-loop biomimetic neuro-robotic prosthesis exploiting neuromorphic hardware is depicted. The pressure events under the prosthetic foot are sensed by the wearable sensors embedded in artificial skin. The stream of information from these sensors is the input for the neuromorphic chip that converts them into bio-inspired neural stimulation patterns resembling natural somatosensory processing. The co-modulations of the neurostimulation parameters and the channels of the implanted neural interfaces will allow the evoking of natural patterns of activation in the residual nervous system of the user. The electrically evoked sensation will be natural and informative allowing for the maximal exploitation of the robotic prosthesis in the sensory-motor loop. A portion of the illustration is adapted from ref. ^42^. Grafica_001 © 2021 by Pietro Comaschi is licensed under CC BY 4.0.

To fulfill this specific design, the neuro-robotic prosthesis should be equipped with multiple wearable sensors able to detect robustly and reliably, in real-time, the interactions with the external world. Signals from multiple sensors have to be streamed and integrated, obtaining a reliable and stable input for the sensory encoding. The neuroprosthesis would require a robotic prosthesis equipped with wearable sensors, a neuromorphic controller chip, and a stimulating system. Sensor information is transmitted to the chip, which transduces them (using the biomimetic encodings, i.e., transfer functions based on natural sensory processing) into instructions for the neural stimulator. In particular, the neuromorphic hardware, embedded in the prosthetic device, would convert the artificial readouts of the sensors into biomimetic neurostimulation patterns. The translated signals are then converted into impulses of current, which are delivered to the residual peripheral nerve through electrodes, implanted directly into the nerve itself. This must be performed in real-time, with latency below 100 ms, to elicit an unperceivable delay for users^140^. This architecture will constitute a neuromorphic sensory neuroprosthesis. At the core of the closed-loop biomimetic prosthesis lies the ability to seamlessly capture, process, and transmit tactile/pressure information. Neuromorphic hardware can process in real-time such information and, by mimicking the intricate neural encoding mechanisms of the somatosensory system, can enable artificial limbs to provide users with natural and informative feedback (Fig. 3).

Then, the intact nervous system does the rest: the signals from the residual nerves are conveyed to the brain of the user, who is able to perceive what happens at the prosthesis and adjust the motor behavior accordingly. The machine and the body are finally connected.

## Future directions and open questions

Humans possess fine motor skills that aid in accurately using and manipulating objects and tools, maintaining balance and walking, or performing any number of highly complex daily activities. In patients with amputations, missing functions can be restored using prostheses. However, dexterous prostheses are highly sophisticated and require the restoration of sensory information to improve the quality of control and acceptability of the patients, enabling a more natural and pleasant experience with the external device.

Analyzing the current state of the field, some recent works showed the first evidence of how biomimetic neural stimulation could be effectively adopted to encode more natural and functional somatosensory feedback in neuroprosthetics. Indeed in PNS, biomimetic stimulation (both for upper- and lower-limb) showed to evoke more natural precepts^8,9^ and more informativeness feedback to accomplish functional tasks^8,9,11^. In CNS, multichannel biomimetic ICMS conveys finely graded force feedback in BCI that more closely approximates the sensitivity conferred by natural touch^7^.

Regarding the engineering challenges, neuromorphic technologies present properties that enable the functional and structural replication of the nervous system, allowing for an ideal solution that integrates with existing neural interfaces, or for the creation of new interfaces in neurorobotic applications.

To make advances in the design of neuromorphic hardware for sensory restoration, and even more in general for the biomimetic approach, it is essential to develop more sophisticated event-based sensors where the sensors are integrated with a silicon model able to generate complex dynamics to resemble biological mechanoreceptors in a compact and low-power design, allows for powerful portable solutions. It is also important to understand how to encode multiple spike trains generated into fewer trains which can then be used to instruct the electric stimulation. This requirement is a consequence of the limited selectivity of the actual sensors, which can be overcome in the future by improving the electrode selectivity and signal bandwidth, more precisely targeting the desired nerve, hence, enhancing discrimination of the elicited sensation.

In addition to the required selectivity, interface stability is a crucial factor for the development of long-term solutions. The implantation of electrodes in human peripheral nerves is associated with biological responses in the nerve due to foreign body reactions (FBRs), causing changes to the properties of nerve–electrode interfaces. Similar FBR was observed for various penetrating electrodes (e.g. Utah array^141^ or polyimide-based electrodes^60^) implanted in human peripheral nerves. The encapsulation of the electrodes and their possible migration are among the theorized causes of poor stability over-time, in particular for evoked sensation location and perceptual threshold. The growth of fibrotic tissue could influence the effectiveness of the neurostimulation (e.g., lower conductivity due to a physical barrier between the electrode and neurons). Interestingly, pharmacological strategies have been proposed to modulate the FBR to neural implants^142^. The use of a collagen coating containing antifibrotic drugs on the intraneural electrode could enable a reduced growth of fibrotic tissue and macrophage infiltration around the implants. The increase in the biocompatibility (i.e., bio-integration and durability) of the device would guarantee the longer-term stability of the implant. Due to the problem of the electrodes’ possible failures and other biological reactions of the nerve (e.g., demyelination, changes in fiber distribution, etc.), constant re-calibration of the neural stimulation would be required. A possible solution to this issue is the exploitation of AI-based algorithms (e.g., Bayesian optimization, reinforcement learning, etc.) able to recalibrate the neurostimulation, identifying the best parameter configuration in a fully automatic and smart way^143^. This combined development of innovative hardware, materials, and software will allow for a future generation of neural interfaces able to provide effective neurostimulation for long-term applications.

Regarding the challenges in interfacing with the nerves, one of the barriers to the success of the approach presented here is related to the ability of evoking a desired spatiotemporal pattern of activation in the targeted neurons. Firstly, even with the latest and most selective available interfaces, the number of neurons activated with each pulse of stimulation is too high. In addition, all the neurons are simultaneously activated causing synchrony that is not present in vivo during natural touch^67^. Indeed, the natural asynchronous activation is driven in part by the probabilistic nature of action potential generation in sensory organs, such as muscle spindles^144^ or retinal cells^145^, and in part by the stochastic nature of synaptic transmission^146^. Recently, interesting approaches exploiting high-frequency electrical stimulation have been proposed to desynchronize neural activity^58,147^. However, the effect of high-frequency stimulation could induce undesired strong sensations, since frequency modulation has an effect on perceived sensation intensity^148^. Another option could be to exploit other techniques in activating the neural tissue, such as optogenetic, and ultrasound stimulation. Indeed, optogenetic stimulation potentially provides the ability to target molecularly defined neuron subtypes, access opsins engendering neural inhibition, and optically recruit axons in a fashion that might mimic natural recruitment^149,150^. In addition, the neuromorphic approach could also be adopted for other sensory prosthesis designs, in which the biomimetic approach has been proven to be effective for improving functional performance (e.g., enhanced speech intelligibility for cochlear implants;^151^ and improved restoration of gaze stability in vestibular prostheses^152^). Thus, the development of prostheses that can account for such pathway-specific heterogeneities will be essential to improving functional outcomes across sensory systems.

Regarding the specific considerations that need to be addressed when generating output signals for PNS stimulation, the safety limits of amplitude, pulse width, and frequency must always be considered to avoid electrode degradation, tissue damage, fast adaptation (Box 5), and sensations that are too strong. Notably, possible interferences with other sensory modalities such as proprioception or motor signals, due to undesired afferent activation, are currently under investigation by research groups working in the field of bidirectional sensory-motor prosthetics^10^.

Finally, the development of fully biomimetic neuroprostheses, able to restore natural and effective sensations, requires a quantitative assessment of the naturalness of this synthetic somatosensory feedback. This assessment represents a further challenge in the development of this type of neuroprosthesis since sensation naturalness is highly subjective. The lack of a bio-signal to objectively quantify the error between the evoked and target sensations makes the development of efficient algorithms very challenging. Perceived naturalness could be influenced by past or present experience, and could be modified with the modulation of stimulation parameters and its related perceived intensity^153^. To this aim, a detailed evaluation is needed, combining both subjective (e.g., questionnaires, free description, psychophysics) and objective measures (e.g., reaction times, neural recordings, performance metrics, learning).

Our perspective informs the design of a future generation of bio-inspired neuroprosthetic devices allowing the artificial conveyance of more complex sensory information with neurostimulation. The approach proposed here could potentially increase, not only the efficacy but also the acceptance of neuroprosthetic devices, improving the quality of life of people with sensory impairments. The use of neurostimulation is also critical to brain–computer interfacing, as well as bioelectronic medicine, in which the electrical stimulation targets the central, or the autonomic, nervous systems, respectively.

Box 5. Neural adaptation to neurostimulation In biological sensory systems, prolonged exposure to a defined stimulus can lead to diminished sensations or their complete extinction. The reason for this behavior lies in a phenomenon called stimulus adaptation, observed across many different species and in several brain sensory areas^222^, e.g., olfactory^223^ and visual^224^ cortex, and consists of a neuronal firing rate decrease in response to a constantly presented stimulus^225^. Adaptation primarily means that the organism detects that a certain monotonous stimulus is no longer of importance and thus can be neglected, to free up resources for a potentially more important change in input. This is a computational mechanism, responsible for separating behaviorally relevant information from the continuous stream of sensory information^226^. Its basis lies in the fact that if there was no habituation or a reduction in responsiveness to a constant and prolonged stimulus, the nervous system would not be able to respond fully to input changes or other stimuli.

Even though this behavior is usually natural and beneficial for the system, when the aim is to artificially restore a sensory channel using neuroprosthetic devices, this phenomenon has to be taken into account. In this application, absolute reliability is necessary to provide meaningful and continuous sensory information to prosthetic users. Indeed, this technology potentially depends upon long-lasting trains of neurostimulation which is potentially compromised by the adaptation of the targeted nerve to neurostimulation^148,227,228^.

To tackle this issue, a deeper understanding of this phenomenon is currently of great interest. For the natural sense of touch, the first stage of sensory adaptation appears at the level of the cutaneous mechanoreceptors^229^. Since in the case of amputation the hand/foot mechanoreceptors are bypassed, the neural stimulation of the proximal residual nerve takes place above the level of the receptors. Thus, in this case, the main factors responsible for sensory adaptation can be summarized as (1) reduction in synaptic transmission (e.g. short-term synaptic depression^230^, presynaptic inhibition^231^ or synaptic depletion^232,233^); (2) conduction failure of the afferent fibers with neurostimulation (i.e., nerve block)^234^ or dorsal root ganglion T-junction filtering^235^; (3) unbalanced activity in excitatory/inhibitory (E/I) networks^236,237^. A conduction block of the nerve can be excluded since this phenomenon typically happens at higher frequencies (>1 kHz^234^). Moreover, adaptation has been observed not only with PNS but also when stimulating the sensory cortex directly (e.g., using intracortical microstimulation^228^).

Interestingly, it is possible to observe important relationships between stimulation parameters and adaptation time variables. For example, varying the amplitude or the frequency of the neurostimulation has a direct effect on the perceived sensation intensity^72,148^, resulting in different adaptation time^227,238^. A possible explanation for this phenomenon can likely be found by analyzing the different spatiotemporal properties of the neurostimulation^148,239^. Modulation of the amplitude is reflected in the recruitment of fiber populations^57,240^, with higher amplitudes activating more fibers. Taking into account the fact that even a small number of activated fibers can deliver meaningful sensory information to the brain^241^, it turns out that with more fibers activated (with higher amplitude), the more time it takes for the sensation to completely disappear due to adaptation. On the other hand, higher frequencies result in increased firing activity, which causes a reduction in synaptic transmission and, eventually, a faster adaptation^148,228^.

Recent findings with vestibular prostheses showed that pulsatile stimulation produced by the prosthesis appears to induce long-term depression at afferent-central neuron synapses in the vestibular nuclei due to the synchrony evoked across the vestibular afferent population—such synchrony is not present during natural head motion^242^. Moreover, there is evidence that multiple sites of plasticity within the vestibular-ocular reflex pathways can rapidly shape motor performance in vivo.

To obtain a comprehensive understanding of the adaptation phenomenon, computational modeling could be a powerful tool to unveil its characteristics and eventually even predict its undesired effects in neuroprosthetic devices^238,243^.
